# Editorial: Nutritional management of childhood obesity and related diseases

**DOI:** 10.3389/fnut.2025.1666185

**Published:** 2025-07-31

**Authors:** Constantinos Giaginis, Sousana K. Papadopoulou

**Affiliations:** ^1^Department of Food Science and Nutrition, School of Environment, University of Aegean, Myrina, Greece; ^2^Department of Nutritional Sciences and Dietetics, School of Health Sciences, International Hellenic University, Thessaloniki, Greece

**Keywords:** childhood obesity, nutritional interventions, dietary pattens, risk factor, metabolic diseases

Childhood obesity constitutes one of the most challenging public health problems of our century due to its epidemic proportions and the related significant morbidity and mortality, increasing also public healthcare costs (1). Alarmingly enough, children with obesity demonstrate a fold-fold higher risk of remaining obese in adulthood (2). Notably, childhood obesity is a major risk factor for many chronic pathological conditions (3). A common risk factor associated with childhood obesity concerns the type of nutrition that the children adopt in their daily life (4). Several nutritional interventions have been proposed with the aim at reducing the prevalence of childhood obesity (5). In this aspect, novel research efforts should be performed, aiming to evaluate potential nutritional interventions, including either specific dietary pattern or foodstuffs ingredients, which may prevent or even co-treat childhood obesity and related diseases.

In view of the above considerations, a population-based study showed that the incidence of hyperuricemia was high in children and adolescents with obesity aged 6–17 years. In addition, this study emphasized that the combination of triponderal mass index and waist-to-height ratio could be applied as a potential early predictor of hyperuricemia risk in children and adolescents with obesity, and especially in girls (Niu et al.). In a cross-section MRI study, adolescents with metabolically healthy obesity showed lower hepatic fat, improved liver markers, and healthier dietary patterns compared to metabolically unhealthy obese peers. This evidence highlights the potential impact of prenatal and lifestyle factors in differentiating metabolic health profiles in adolescents affected by obesity (Moran-Lev et al.). Another cross-sectional study from NHANES 2011–2016 was designed to evaluate the association between the composite dietary antioxidant index (CDAI) and the prevalence of overweight or obesity among children and adolescents aged 6–19 years in the United States. This survey developed a modified CDAI, comprising of vitamins A, C, E, carotenoids, and zinc, and identified a consistent negative association between modified CDAI and overweight/obesity risk, regardless of energy adjustment method. This evidence suggests that a diet rich in antioxidants may exert a protective role in preventing obesity in adolescents aged 12–19 years (Chen and Shi).

A comprehensive national cross-sectional study in preschool children in Jordan was designed to assess feeding practices of infants and young children, determining the frequency of consuming micronutrient-rich foods, evaluating causes of anemia, assessing the health status of specific subgroups, and comparing findings to the previous 2010 national study (Barham et al.). In the previous 2010 national study, high rates of iron and vitamin D deficiencies among preschool children, with about 20% experiencing vitamin A deficiencies were recorded. The 2019 national study highlights ongoing nutritional challenges among Jordanian preschoolers. Although severe anemia was rare, 11% were anemic, and 22.4% had iron deficiency, including 5% with iron deficiency anemia. Vitamin D deficiency affected 22.9%, impacting growth and immunity. While stunting and wasting improved, childhood overweight and obesity rates remained steady. Anemia decreased, but iron deficiency rose by 7%. Despite reduced vitamin A deficiency, stable iron deficiency anemia rates indicate ongoing concerns. Overall, this study supported evidence that undernutrition is uncommon, but vitamin D and iron deficiencies, along with childhood obesity, need sustained attention and targeted interventions to improve children's health in Jordan (Barham et al.). A retrospective study analyzed clinical data from 159 school-aged children to investigate the potential association between vitamin D deficiency and childhood obesity rates, and its impact on serum calcium, alkaline phosphatase, and bone age in children. This study showed that the 25-hydroxyvitamin D3 [25(OH)D3] deficiency cohort exhibited significantly higher body mass index (BMI), total cholesterol (TC), triglycerides (TG), and alkaline phosphatase (ALP) levels, with lower Calcium (Ca) levels and delayed bone age compared to the normal group. These findings supported evidence that 25(OH)D_3_ deficiency is strongly associated with obesity in school-aged children and may negatively affect normal skeletal development. This study also suggests that regular monitoring of 25(OH)D_3_ levels in school-aged children is essential for ensuring proper growth and development, especially in those at risk for obesity (Xu et al.).

Another study explored the potential associations of micronutrients and lipids with prediabetes, glycemic parameters, and glycemic indices among 1,520 adolescent girls aged 16–18 years of the DERVAN cohort study from rural India. This study found a substantial deficiency of micronutrients and an absence of dyslipidemia. Moreover, this study highlights the need for lipid and micronutrient-based interventions in adolescence to improve glycemic outcomes, supporting that maintaining adequate storage of not only micronutrients but also lipids in adolescent girls is likely to reduce diabetes risk in adulthood (Patil et al.). A cross-sectional study was designed to assess the synergistic impact of Mediterranean diet, lifestyle and technology on glycemic control in 112 children with type 1 diabetes (T1D) from Gran Canaria (median age 12 years). This study showed that Mediterranean diet compliance, insulin delivery methods, age, and number of years with T1D could be important factors to consider in the management of T1D in children (Nóvoa-Medina et al.).

A multicenter cross-sectional nutritional and health surveillance study of a nationally representative sample of urban populations from eight Latin American countries aimed to explore the potential associations between the energy imbalance gap (EIG) and sociodemographic and anthropometric variables. A total of 680 adolescents aged 15–18 were included in this study, while the estimation of energy intake was based on two non-consecutive 24-h dietary recalls. This study supported evidence that sex and BMI were associated with EIG in adolescents from Latin America (Hernandez et al.). An institutional-based cross-sectional study design was conducted among 366 mothers with children aged 6–23 months in Ethiopia. This survey was designed to explore complementary food hygiene practices and their associated factors. It was found that the prevalence of complementary food hygiene practices was poor. This survey supported evidence that healthcare professionals should promote starting breastfeeding at the age of 6 months. In addition, media companies ought to make an effort to create a positive social and cultural environment that encourages complementary feeding practices for young children (Addis et al.). A cross-sectional study aimed to assess Online food delivery applications (OFDA) usage trends among adolescent users in the United Arab Emirates (UAE), focusing on their perceptions of healthy food options and food safety (*n* = 532). Most participants used OFDAs weekly (65.4%), favoring fast food (85.7%). Factors like appearance and price drove food choices (65.0%), while taste and cost hindered healthy food orders. Younger and frequent users had lower scores for perceiving healthy food, while seeking healthy options was associated with higher scores. Females and those seeking healthy food showed higher food safety scores. The study suggests tailored interventions to promote healthier choices and improve food safety perceptions among adolescents using OFDAs in the UAE (Saleh et al.).

Conclusively, the currently available research reinforces the urgent demand for the development and implementation of well-organized public strategies and policies that could inform the future parent about the beneficial effects of diverse nutritional interventions at the early stages of their children life in combination with other lifestyle factors, e.g., physical activity, mental health, metabolic disturbances, against childhood overweight, and obesity. Most of the currently available studies have a cross-sectional design, which cannot support causality effects. In this aspect, the performance of longitudinal studies is highly recommended.
